# Use of Chlorothiazide in the Management of Central Diabetes Insipidus in Early Infancy

**DOI:** 10.1155/2017/2407028

**Published:** 2017-05-03

**Authors:** Manish Raisingani, Resmy Palliyil Gopi, Bina Shah

**Affiliations:** Department of Pediatrics, Division of Pediatric Endocrinology, New York University School of Medicine, New York, NY, USA

## Abstract

Management of central diabetes insipidus in infancy is challenging. The various forms of desmopressin, oral, subcutaneous, and intranasal, have variability in the duration of action. Infants consume most of their calories as liquids which with desmopressin puts them at risk for hyponatremia and seizures. There are few cases reporting chlorothiazide as a temporizing measure for central diabetes insipidus in infancy. A male infant presented on day of life 30 with holoprosencephaly, cleft lip and palate, and poor weight gain to endocrine clinic. Biochemical tests and urine output were consistent with central diabetes insipidus. The patient required approximately 2.5 times the normal fluid intake to keep up with the urine output. Patient was started on low renal solute load formula and oral chlorothiazide. There were normalization of serum sodium, decrease in fluid intake close to 1.3 times the normal, and improved urine output. There were no episodes of hyponatremia/hypernatremia inpatient. The patient had 2 episodes of hypernatremia in the first year of life resolving with few hours of hydration. Oral chlorothiazide is a potential bridging agent for treatment of central DI along with low renal solute load formula in early infancy. It can help achieve adequate control of DI without wide serum sodium fluctuations.

## 1. Introduction

Central diabetes insipidus (DI) refers to the inability to conserve free water and is most commonly caused by hypothalamic/posterior pituitary lesions, resulting in impaired arginine vasopressin (AVP) production and release. In the neonatal period it may be associated with midline brain anatomic abnormalities such as septooptic dysplasia with agenesis of the corpus callosum, Kabuki syndrome, holoprosencephaly, and familial pituitary hypoplasia with absent stalk [1–4]. Infections involving the base of the brain, such as meningococcal, cryptococcal, listeria, and toxoplasmosis meningitis, congenital cytomegalovirus infection, and nonspecific inflammatory disease of the brain, can cause central diabetes insipidus in infancy [5–9]. Due to the deficiency of AVP, patients with central DI are unable to concentrate their urine and have a very low urine osmolality. They have to consume enormous amounts of water to keep up with the urine output. With fluid management alone these children can develop nonobstructive hydroureteronephrosis, bladder wall thickening and trabeculations, overflow incontinence, and impaired renal function requiring a drainage procedure [10]. Hence, vasopressin analogues have been used to help decrease urine output and increase urine concentrating ability.

One situation where vasopressin analogues may not always be readily used to manage central DI is infancy. The various forms of vasopressin analogues, oral, subcutaneous, or intranasal, have variability in duration of action with no established safety and efficacy data in early infancy [11–13]. Their use is associated with wide fluctuations in serum sodium (Na) levels. In addition, infants have high obligate fluid requirement (3 L/m2/day) and consume most of the calories as liquids. This high fluid intake along with desmopressin (DDAVP) puts them at an increased risk for hyponatremia and seizures. There have been a few case reports of managing central DI in early infancy using low renal solute load formula and chlorothiazide [14–16]. These patients were transitioned to DDAVP when the baby got older and started consuming more solid food. We report the case of a month-old baby with holoprosencephaly and central diabetes insipidus managed with oral chlorothiazide during infancy.

## 2. Case Report

A male infant presented on day of life (DOL) 30 to the endocrine clinic with holoprosencephaly, cleft lip, cleft palate, and poor weight gain. Baby was born at an outside hospital and transferred to our facility due to congenital defects of cleft lip and cleft palate. Baby was born, full term NSVD, with APGAR scores of 9 and 9 at 1 and 5 minutes. Pregnancy was complicated by gestational diabetes controlled with insulin. Immediate newborn course was complicated by polycythemia which resolved with partial exchange transfusion. Baby had normal Na levels 144 mmol/L on DOL 2. Baby was transferred on DOL 15 for surgical evaluation and management to our facility. MRI of the brain revealed corpus callosum dysgenesis with partial absence of falx cerebri as well as mild colpocephaly and possible lobar holoprosencephaly. The baby's weight on DOL 15 was 8.5% below the birth weight. He had an uneventful course except for feeding issues due to cleft lip and cleft palate. Baby's feeding and weight gain improved on a high calorie formula and the baby was discharged on DOL 24 from the NICU. After the initial normal electrolytes, they were not repeated during the NICU hospitalization. Baby was referred to and seen in pediatric endocrine clinic on DOL 30 to evaluate pituitary function and was again noted to have poor weight gain and had gained only 115 grams over the birth weight. Serum Na level was 163 mmol/L and baby was admitted to the pediatrics unit.

**Figure 1:**
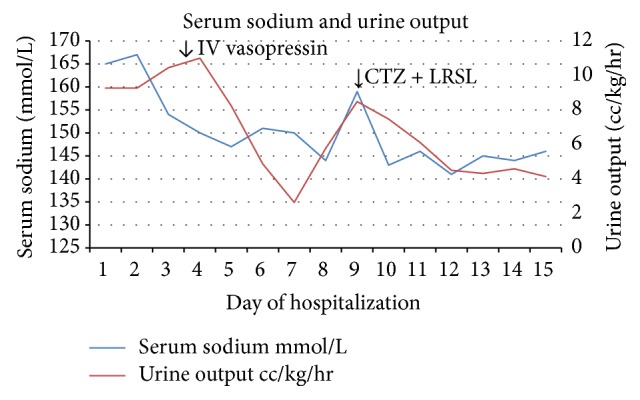
Serum sodium levels and urine output during hospitalization. CTZ: chlorothiazide; LRSL: low renal solute load formula.

Baby was noted to have a urine output of 8–11 cc/kg/hr and required huge amounts of PO and IV fluids to keep up with the losses. Urine specific gravity (<1.005) and urine osmolality (~100 mOsm/kg) were consistent with central diabetes insipidus. Baby was started on IV vasopressin and IV fluids. On this management urine output and serum Na levels normalized. Baby had a gastrostomy tube placement on DOL 35 to provide adequate fluids/nutrition. Vasopressin drip was discontinued on DOL 37 and low renal solute load formula (RSL) was started the same day. Oral chlorothiazide was started on DOL 39. There was improvement in urine output (4-5 cc/kg/hr) and serum Na levels normalized (Figure 1) with improved weight gain and the patient was discharged home on DOL 44. The treatment was initiated with a chlorothiazide dose of 2 mg/kg/day and it was titrated based on serum Na levels to 6 mg/kg/day.

Results of the rest of the pituitary workup were normal: free T4 1.30 ng/dL (16.73 pmol/L), IGF-1 38 ng/mL (4.97 nmol/L), and random cortisol 13.7 mcg/dL (378 nmol/L). Genetic testing revealed a normal male karyotype with no abnormality seen on microarray. Repeat pituitary function testing has been normal with random cortisol 13.6 mcg/dL (375 nmol/L), IGF-1 levels 55–69 ng/mL (7.19–9.02 nmol/L), and free T4 levels 1.08–1.16 ng/dL (13.9–14.93 pmol/L).

The baby has been followed up in the outpatient clinic for 15 months with maintenance of Na levels near the higher end of normal. Baby had an admission at 7 months of age for respiratory distress and poor oral intake. The baby was not feeding well and mother was not using the gastrostomy tube. Mother also missed one dose of chlorothiazide. In the hospital, the baby was given home feeds via gastrostomy tube and IV fluids for a few hours, with which the baby did well and was discharged home. Since the baby is unable to take solid food due to cleft lip and palate, there has been slow weight gain. Pediasure which has a high renal solute load was added to the regimen at 11 months of age which led to an episode of hypernatremia (serum Na 162 mmol/L). He was admitted and started on free water 15–20 cc with each feed and the episode resolved. The baby is being evaluated to undergo surgery by ENT for cleft lip and cleft palate after which he may be able to take solid food. The plan is to switch to oral DDAVP once the baby is able to consume most of the calories as solid food.

## 3. Discussion

The management of central DI in infancy is very challenging due to very few safe treatment options. There have been problems reported in literature with use of DDAVP in infancy as listed in Table 1. At one center, DDAVP was administered intranasally to four infants (0.5–2.5 *μ*g per dose) and subcutaneously to two infants (0.125 *μ*g per dose) who were diagnosed with central diabetes insipidus in infancy [12]. Three of six infants had at least one episode of hyponatremia (serum sodium < 130 mmol/L). All infants had at least one episode of hypernatremia with serum Na > 160 mmol/L. Three infants required hospitalization for treatment of hyponatremia or hypernatremia (174–189 mmol/L). Other investigators have also reported variability in duration of action with using nasal and oral forms of DDAVP [11, 13, 17]. Rivas-Crespo et al. also reported an infant on intranasal DDAVP who had episodes of hypernatremia whenever he had a cold due to poor absorption and ultimately developed central myelinolysis during one of the episodes when serum Na was 189 mmol/L [18]. Blanco et al. had some success using subcutaneous form of DDAVP, although the dose needs to be carefully titrated. There were episodes of hyponatremia in five of the six patients treated with subcutaneous DDAVP, although it was asymptomatic and in most of the cases it took place initially when the dose was being adjusted. This dose needs to be carefully titrated, preferably in an inpatient setting [19]. There are some cases in literature reporting use of alternative treatments like carbamazepine [20], chlorpropamide [21, 22], and clofibrate [21, 23] for management of central diabetes insipidus. There is lack of data regarding their use in central diabetes insipidus in infancy and a concern for serious side effects like hypoglycemia [24] or increased mortality [20].

There have been a few case reports describing the use of chlorothiazide or hydrochlorothiazide along with low renal solute formula for the management of central diabetes insipidus in infancy as listed in Table 1. Rivkees et al. described successful treatment of central DI in 5 infants with chlorothiazide and using low RSL, breast milk, or diluting formula with water. The infants were transitioned to DDAVP between 6 and 18 months of age. Similarly, Abraham et al. described 4 cases with central DI diagnosed before 10 days of age. These infants were managed on hydrochlorothiazide and low RSL formula with transition to oral DDAVP between 3 and 12 months of age. Chaudhary et al. used hydrochlorothiazide with diluted formula to control serum sodium levels in an infant with holoprosencephaly and central DI.

Chlorothiazide is a drug used in the management of nephrogenic DI. The mechanism of action of chlorothiazide is not exactly known. The most widely accepted hypothesis suggests that the antidiuretic action of thiazides is secondary to increased renal sodium excretion. The renal sodium loss causes extracellular volume contraction leading to lowered GFR and increased proximal tubular sodium and water reabsorption. Hence, less water and solutes are delivered to the distal tubule and collecting duct and less are lost as urine [25, 26]. It is unclear what the mechanism of action is in central DI but is believed to be due to the same process as described above. In addition using a low RSL formula (Sim PM 60/40 in our case) decreases the solute load on the kidney and hence causes a decrease in the obligate urine output.

Renal solute load (RSL) refers to all solutes of endogenous or dietary origin that require excretion by the kidneys. Potential renal solute load (PRSL) refers to solutes of dietary origin that would need to be excreted in the urine if none were diverted into synthesis of new tissue and none were lost through nonrenal routes. It is dependent on the nitrogen, sodium, chloride, potassium, and phosphorus content of the formula [27, 28]. Low renal solute load formula helps decrease urine output as the amount of solutes that need to be excreted by the kidney is reduced. Sim PM 60/40 and breastmilk both have a 20–30% lower renal solute load than other commercially available formulas [16]. Hence substituting regular formula with low RSL formula or breast milk may help decrease urine output in patients with central DI.

In conclusion oral chlorothiazide may be a potential bridging agent for treatment of central DI along with low RSL formula, when diet is mostly fluids in early infancy. It can help achieve adequate control of DI without risks of wide serum sodium fluctuations. Transition to DDAVP should occur when these infants consume more solid food. More studies should be done evaluating chlorothiazide use for central diabetes insipidus in infancy.

## Figures and Tables

**Table 1:** Summary of cases of central diabetes insipidus in infancy.

Author	Age at diagnosis in days	Cause of DI	Treatment	Age at transition in months to DDAVP	Complications	Number of hyponatremia episodes	Number of hypernatremia episodes
Rivkees et al., 5 infants	Early infancy	N/A	BM or LRSL + free water + CTZ	6–18	N/A	0	1 episode
Abraham et al.	<7	Septooptic dysplasia	LRSL + HCTZ	12	FTT	0	0
Abraham et al.	<7	Septooptic dysplasia	LRSL + HCTZ	3	Acute gastroenteritis with low K, difficulty in maintaining sodium	0	0
Abraham et al.	<7	Septooptic dysplasia	LRSL + HCTZ	6	Failure to thrive	0	0
Abraham et al.	<7	Holoprosencephaly	LRSL + HCTZ	12	FTT, hypernatremia	0	0
Chaudhary et al.	10	Holoprosencephaly	Dilution of formula + HCTZ	N/A	N/A	0	0
Pogacar et al., 6 infants	Early infancy	Holoprosencephaly Trauma, panhypopituitarism, and meningitis	DDAVP: intranasal, subcutaneous	N/A	3 infants hospitalized for hypo- or hypernatremia, 1 death due to hyponatremic seizure	At least 5	At least 3
Blanco et al., 10 infants	Infancy	Septooptic dysplasia, holoprosencephaly, congenital nasal piriform sinus stenosis, group Β streptococcal meningitis, and congenital diabetes insipidus	DDAVP: intranasal, 4 patients DDAVP: subcutaneous, 6 patients	N/A	N/A	9 out of 10 patients had hyponatremia	Inpatient, 9 episodes; outpatient, 5 episodes
Yarber et al.	<7 days	NA	DDAVP: subcutaneous	N/A	N/A	N/A	N/A
Rivas-Crespo et al.	28 days	Neonatal hemorrhagic shock	Subcutaneous + intranasal	N/A	Central myelinolysis at age 3 due to poor absorption of intranasal DDAVP	N/A	Multiple hypernatremia on intranasal DDAVP

DDAVP: desmopressin, FTT: failure to thrive, BM: breast milk, LRSL: low renal solute load formula, CTZ: chlorothiazide, HCTZ: hydrochlorothiazide, and N/A: not available.

## Additional Points

This article discusses challenges faced in the management of central diabetes insipidus in infancy.
